# Effectiveness and Safety of Using Chatbots to Improve Mental Health: Systematic Review and Meta-Analysis

**DOI:** 10.2196/16021

**Published:** 2020-07-13

**Authors:** Alaa Ali Abd-Alrazaq, Asma Rababeh, Mohannad Alajlani, Bridgette M Bewick, Mowafa Househ

**Affiliations:** 1 College of Science and Engineering Hamad Bin Khalifa University Doha Qatar; 2 Jordan Health Aid Society International Amman Jordan; 3 Institute of Digital Healthcare University of Warwick Warwick United Kingdom; 4 Leeds Institute of Health Sciences School of Medicine University of Leeds Leeds United Kingdom

**Keywords:** chatbots, conversational agents, mental health, mental disorders, depression, anxiety, effectiveness, safety

## Abstract

**Background:**

The global shortage of mental health workers has prompted the utilization of technological advancements, such as chatbots, to meet the needs of people with mental health conditions. Chatbots are systems that are able to converse and interact with human users using spoken, written, and visual language. While numerous studies have assessed the effectiveness and safety of using chatbots in mental health, no reviews have pooled the results of those studies.

**Objective:**

This study aimed to assess the effectiveness and safety of using chatbots to improve mental health through summarizing and pooling the results of previous studies.

**Methods:**

A systematic review was carried out to achieve this objective. The search sources were 7 bibliographic databases (eg, MEDLINE, EMBASE, PsycINFO), the search engine “Google Scholar,” and backward and forward reference list checking of the included studies and relevant reviews. Two reviewers independently selected the studies, extracted data from the included studies, and assessed the risk of bias. Data extracted from studies were synthesized using narrative and statistical methods, as appropriate.

**Results:**

Of 1048 citations retrieved, we identified 12 studies examining the effect of using chatbots on 8 outcomes. Weak evidence demonstrated that chatbots were effective in improving depression, distress, stress, and acrophobia. In contrast, according to similar evidence, there was no statistically significant effect of using chatbots on subjective psychological wellbeing. Results were conflicting regarding the effect of chatbots on the severity of anxiety and positive and negative affect. Only two studies assessed the safety of chatbots and concluded that they are safe in mental health, as no adverse events or harms were reported.

**Conclusions:**

Chatbots have the potential to improve mental health. However, the evidence in this review was not sufficient to definitely conclude this due to lack of evidence that their effect is clinically important, a lack of studies assessing each outcome, high risk of bias in those studies, and conflicting results for some outcomes. Further studies are required to draw solid conclusions about the effectiveness and safety of chatbots.

**Trial Registration:**

PROSPERO International Prospective Register of Systematic Reviews CRD42019141219; https://www.crd.york.ac.uk/prospero/display_record.php?ID=CRD42019141219

## Introduction

### Background

Mental illness is a growing public health concern worldwide [1]. One in 4 adults and 1 in 10 children are likely to be affected by mental health problems annually [2]. Mental illness has a significant impact on the lives of millions of people and a profound impact on the community and economy. Mental disorders impair quality of life and are considered one of the most common causes of disability [3]. Mental disorders are predicted to cost $16 trillion globally between 2011 and 2030 due to lost labor and capital output [4].

There is a shortage of mental health human resources, poor funding, and mental health illiteracy globally [5,6]. This lack of resources is especially evident in low-income and middle-income countries where there are 0.1 psychiatrists per 1,000,000 people [7], compared to 90 psychiatrists per 1,000,000 people in high-income countries [8]. According to the World Health Organization, mental health services reach 15% and 45% of those in need in developing and developed countries, respectively [9]. This could be a major factor contributing to the increase in suicidal behavior in recent decades [10].

The demand for better mental health services has increased, and meeting these demands has become increasingly difficult and costly due to a lack of resources [4]. Therefore, new solutions are needed to compensate for the deficiency of resources and promote patient self-care [4]. Distance can impede the reach of traditional mental health services to populations in remote areas in both high-income and low-income countries. Technology-based treatment, such as mobile apps, can overcome most of these barriers and engage hard-to-reach populations [11]. A World Health Organization survey of 15,000 apps revealed that 29% focus on mental health diagnosis or support [10].

One technology that offers a partial solution to the lack of capacity within the global mental health workforce is mobile apps. They have the potential to improve the quality and accessibility of mental health [12]. Chatbots are one of the main mobile apps used for mental health [13]. Chatbots, also known as conversational agents, conversational bots, and chatterbots, are computer programs able to converse and interact with human users [5,14]. Chatbots use spoken, written, and visual languages [5,14]. The use of chatbots has grown tremendously over the last decade and has become pervasive in fields such as mental health [13]. It is expected that chatbots will make a positive contribution to addressing the shortfall of mental health care [15]. Chatbots can facilitate interactions with those who are reluctant to seek mental health advice due to stigmatization [5] and allow more conversational flexibility [16].

### Research Problem and Aim

Adoption of new technology, especially those heavily related to artificial intelligence and machine learning, relies on first ascertaining the levels of safety and effectiveness [17]. There has been a steady rise in the number of studies assessing the effectiveness and safety of using chatbots for mental health [5]. There is a need to critically evaluate and statistically combine findings to inform policy and practice. Previously conducted reviews [5,18,19] did not assess the effectiveness and safety of chatbots in mental health. Accordingly, the current systematic review aimed to assess the effectiveness and safety of using chatbots in mental health through summarizing and pooling the results of previous studies. The review question is “what is the effectiveness and safety of using chatbots for improving mental health?”

## Methods

### Overview

A systematic review of the literature was conducted to accomplish the objective. This review is reported in line with the Preferred Reporting Items for Systematic Reviews and Meta-Analyses statement (Multimedia Appendix 1) [20]. The protocol for this systematic review is registered at PROSPERO (number CRD42019141219).

### Search Strategy

#### Search Sources

The following bibliographic databases were searched in this review: MEDLINE, EMBASE, PsycINFO, IEEE Xplore, ACM Digital Library, Scopus, and Cochrane Central Register of Controlled Trials. The search engine “Google Scholar” was also searched. As Google Scholar retrieved a large number of studies ordered by their relevance to the search topic, we screened the first 100 hits (10 pages). The search started on the June 8, 2019 and finished on June 11, 2019. We carried out backward reference list checking, where reference lists of the included studies and reviews were screened for further studies of relevance to the review. In addition, we conducted forward reference list checking, where we used the “cited by” function available in Google Scholar to identify studies that cited the included studies.

#### Search Terms

Search terms in this review were related to population (eg, mental disorder, mood disorder, and anxiety disorder) and intervention (eg, conversational agent, chatbot, chatterbot, and virtual agent). The search terms were derived from previous reviews and informatics experts interested in mental health issues [13]. Further, search terms related to mental disorders were derived from the Medical Subject Headings index in MEDLINE. The search strings utilized for searching each bibliographic database are shown in Multimedia Appendix 2.

### Study Eligibility Criteria

The population of interest was individuals who use chatbots for their mental health, but not physicians or caregivers who use chatbots for their patients. Eligible interventions were chatbots operating as standalone software or via a web browser. Chatbots that were integrated into robotics, serious games, SMS, or telephone systems were excluded. The current review also excluded chatbots that relied on human-operator generated dialogue. There were no restrictions regarding the type of dialogue initiative (ie use, system, mixed) and input and output modality (ie spoken, visual, written). There were no limitations related to the comparator (eg, information, waiting list, usual care). This review focused on any outcome related to effectiveness (eg, severity or frequency of any mental disorders and psychological wellbeing) or safety (eg, adverse events, deaths, admissions to psychiatric settings) of chatbots. Regarding the study design, we included only randomized controlled trials (RCTs) and quasiexperiments. The review included peer-reviewed articles, dissertations, conference proceedings, and reports. The review excluded reviews, conference abstracts, proposals, and editorials. Only studies written in English were included in the review. There were no restrictions regarding study setting, year of publication, and country of publication.

### Study Selection

Two steps were followed for selecting studies. First, the titles and abstracts of all retrieved studies were screened independently by two reviewers (AA, MA). Second, the full texts of studies included from the first step were read independently by the same reviewers. Any disagreements between the reviewers were resolved by discussion or by consulting a third reviewer (MH). Cohen κ [21] was calculated to assess interrater agreement between reviewers, which was 0.85 and 0.89 in the first and second step of the selection process, respectively, indicating a very good level of agreement [22].

### Data Extraction

Before extracting data, we developed a data extraction form and piloted it using three included studies to conduct a systematic and precise extraction of data (Multimedia Appendix 3). Two reviewers (AA, MA) independently extracted data from the included studies, and disagreements were resolved by discussion or by consulting the third reviewer (MH). Interrater agreement between the reviewers was very good (Cohen κ=0.84) [22].

### Assessment of Risk of Bias

Two Cochrane tools were used to assess the risk of bias in the included studies. Risk of bias in RCTs was assessed using the Risk of Bias 2 (RoB 2) tool [23], and risk of bias in quasi-experiments was examined using the Risk Of Bias In Non-randomized Studies – of Interventions (ROBINS-I) tool [24]. The results of the risk of bias are presented as a graph showing the reviewers’ judgments about each “risk of bias” domain. Further, they are presented as a figure showing the reviewers’ judgments about each “risk of bias” domain for each included study. Two reviewers (AA, AR) independently assessed the risk of bias, and disagreements were resolved by discussion or by consulting the third reviewer (MH). Interrater agreement between the reviewers was good (Cohen κ=0.75) [22].

### Data Synthesis

Data extracted from studies were synthesized using narrative and statistical methods. The statistical approach was used when there was more than one RCT for a certain outcome and the study reported enough data for the analysis. Where statistical findings were not available, a narrative approach was used to synthesize the data. Findings of studies were grouped and synthesized according to the measured outcome.

Statistical analysis was carried out using Review Manager (RevMan 5.3). As all extracted data were continuous, the effect of each trial and the overall effect were measured using either the mean difference (MD) or the standardized mean difference (SMD). To be more precise, when the outcome was measured using the same method between studies, the MD was utilized. The SMD was used when, between studies, the outcome was assessed using different measurement tools. A random-effects model was used for combining results because there was clinical heterogeneity between studies in terms of population (eg, clinical versus nonclinical samples), intervention (eg, rule-based versus artificial intelligence chatbots), and comparator (eg, treatment as usual versus information).

When there was a statistically significant difference between groups, we assessed how this difference was clinically important. A minimal clinically important difference refers to the smallest change in a measured outcome that a patient would deem as worthy and significant and which mandates a change in a patient’s treatment [25]. Boundaries of a minimal clinically important difference for each outcome were calculated as ±0.5 times the SD of the control arms of the studies at baseline.

Clinical heterogeneity of the meta-analyzed trials was assessed by checking their participants, interventions, comparators, and measured outcomes. Statistical heterogeneity was assessed by calculating the statistical significance of heterogeneity (chi-square *P* value) and I^2^. A chi-square *P* value >.05 indicates that the studies are homogenous [26]. I^2^ was used to quantify the heterogeneity of studies, where I^2^ of 0%-40%, 30%-60%, 50%-90%, and 75%-100% represents unimportant, moderate, substantial, and considerable heterogeneity, respectively [26].

When the evidence was synthesized statistically, the overall quality of that evidence was assessed using the Grading of Recommendations Assessment, Development and Evaluation [17]. Two reviewers (AA & AR) assessed the quality of the evidence, and disagreements were resolved by discussion or by consulting the third reviewer (MH). There was considerable interrater agreement between the reviewers (Cohen κ= 0.86) [22].

## Results

### Search Results

**Figure 1:**
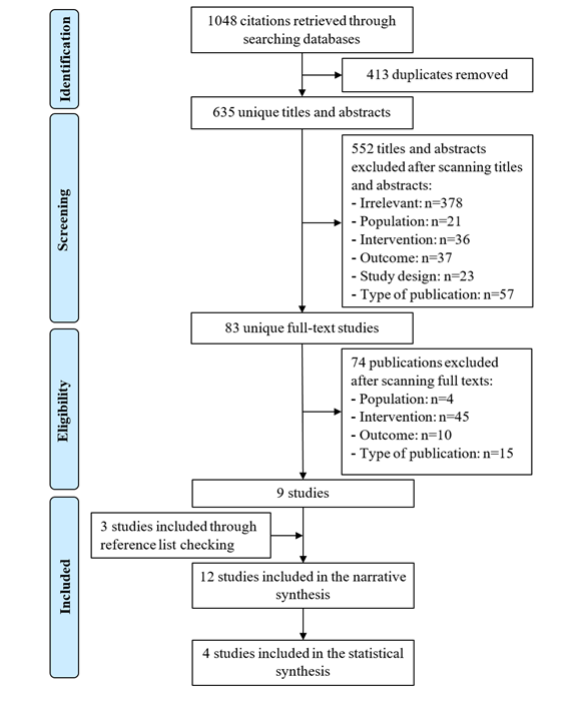
Flow chart of the study selection process.

The search retrieved 1048 citations (Figure 1). After removing 413 duplicates, 635 unique titles and abstracts remained. By screening those titles and abstracts, 552 citations were excluded. Of the remaining 83 studies, 9 studies were included after reading the full text. Two additional studies were identified from forward reference list checking, and one study was identified by backward reference list checking. Overall, 12 studies were included in the narrative synthesis, but only 4 of those studies were meta-analyzed.

### Description of Included Studies

As shown in Table 1, half of the studies (6/12) were RCTs, while the other half were quasiexperimental. Two-thirds of studies (8/12) were journal articles. Studies were conducted in more than 11 countries. Studies were published between 2015 and 2018. The majority of studies was published in 2018 (7/12). The sample size was <100 in 6 studies (6/12, 50%), and sample sizes ranged from 10 to 454 participants, with a median of 71.5 participants. The age of participants was reported in 10 studies; the mean age of participants in those studies was 31.3 years. The sex of participants was reported in 9 studies; the mean percentage of male participants in those studies was 35%. Half of the studies (6/12) recruited nonclinical samples. Participants were recruited from either community (6/12), educational (4/12), or clinical (3/12) settings. The characteristics of each included study are shown in Multimedia Appendix 4.

**Table 1:** Characteristics of the included studies (n=12).

Characteristics			Number of studies
**Study design**			
	Quasiexperiment	6	
	Randomized controlled trial	6	
**Type of publication**			
	Journal article	8	
	Conference proceedings	3	
	Thesis	1	
**Country**			
	United States	4	
	Japan	1	
	Sweden	1	
	Turkey	1	
	Australia	1	
	United Kingdom	1	
	China	1	
	Romania, Spain, and Scotland	1	
	Global population	1	
**Year of publication**			
	2018	7	
	2017	2	
	2016	1	
	2015	2	
**Sample size**			
	<100	6	
	100-200	3	
	>200	1	
Age (years), mean (range)^a^			31.3 (22-45)
Sex (male), %^b^			35
**Sample type**			
	Clinical sample	6	
	Nonclinical sample	6	
**Setting^c^**			
	Community	6	
	Educational	4	
	Clinical	3	
**Intervention purpose**			
	Therapy	10	
	Self-management	2	
**Intervention platform**			
	Web-based	6	
	Standalone software	6	
**Intervention response generation**			
	Rule-based	8	
	Artificial intelligence	4	
**Intervention dialogue initiative**			
	Chatbot	9	
	Both	3	
**Intervention input modality**			
	Written	9	
	Spoken	2	
	Written and spoken	1	
**Intervention output modality**			
	Written	6	
	Written, spoken, and visual	3	
	Spoken and visual	2	
	Written and visual	1	
**Embodiment**			
	Yes	6	
	No	6	
**Targeted disorders^d^**			
	Depression	7	
	Anxiety	4	
	Any mental disorder	3	
	Acrophobia	1	
	Stress	1	
**Comparator**			
	Pretest vs posttest		
	No intervention	4	
	Education	3	
	High users vs low users	1	
**Measured outcomes^e^**			
	Severity of depression	6	
	Psychological wellbeing	3	
	Severity of anxiety	3	
	Positive and negative affect	2	
	Distress	2	
	Stress	2	
	Safety	2	
	Severity of acrophobia	1	
**Measures^f^**			
	PHQ-9^g^	4	
	GAD-7^h^	2	
	PANAS^i^	2	
	K10^j^	2	
	PSS-10^k^	2	
	AQ^l^	1	
	HAD-S^m^	1	
	OASIS^n^	1	
	WHO-5-J^o^	1	
	HIQ^p^	1	
	BDI-2^q^	1	
	Adverse events	2	
**Follow-up period^r^**			
	2 weeks	6	
	4 weeks	6	
	6 weeks	1	
	12 weeks	1	

^a^Mean age was reported in 10 studies.

^b^Sex was reported in 9 studies.

^c^Numbers do not add up as one study recruited the sample from more than one setting.

^d^Numbers do not add up as 4 chatbots focused on both depression and anxiety.

^e^Numbers do not add up as most studies assessed more than one outcome.

^f^Numbers do not add up as some studies used more than one tool to assess a single outcome and several studies have more than one outcome.

^g^PHQ-9: Patient Health Questionnaire.

^h^GAD-7: Generalized Anxiety Disorder scale.

^i^PANAS: Positive and Negative Affect Schedule.

^j^K10: Kessler Psychological Distress Scale.

^k^PSS-10: Perceived Stress Scale.

^l^AQ: Acrophobia Questionnaire.

^m^HAD-S: Hospital Anxiety and Depression Scale.

^n^OASIS: Overall Anxiety Severity and Impairment Scale.

^o^WHO-5-J: World Health Organization-5 Well-Being Index.

^p^HIQ: Heights Interpretation Questionnaire.

^q^BDI-2: Beck Depression Inventory II.

^r^Numbers do not add up as two studies assessed outcomes at 2 different points of time.

The included studies investigated the effect of 11 different chatbots. In most studies (10/12) chatbots were used for delivering therapy (Table 1). Chatbots were implemented using standalone software (6/12, 50%) and in web-based platforms (6/12, 50%). Chatbot responses were based on predefined rules or decision trees (rule-based) in two-thirds of studies (8/12). Chatbots in the remaining one-third of studies (4/12) utilized machine learning and natural language processing (artificial intelligence) to understand users’ replies and generate responses. Chatbots led and controlled the conversation in 75% (9/12) of the studies. Users could interact with the chatbots using only written language via keyboards and mouse (9/12), only spoken language via microphones (2/12), or a combination of written and spoken languages (1/12). Chatbots used the following modalities to interact with users: only written language via text on the screen (6/12); a combination of written, spoken (via speakers), and visual languages (via embodiment) (3/12); a combination of spoken and visual languages (2/12); and a combination of written and visual languages (1/12). In half of the studies, chatbots contained virtual representations (eg, avatar). Chatbots in 58% of the studies targeted depression (7/12). Multimedia Appendix 5 shows the characteristics of the intervention in each study.

There was no comparator in the 4 one-arm quasiexperiments; these quasiexperimental studies assessed outcomes before and after the intervention (Table 1). In 4 additional studies, an intervention was not provided to the control group. In 3 additional studies, chatbots were compared with providing information or education. In the remaining study, the comparison was between high users (more engaged app users) and low users (less engaged app users). The most common outcome assessed by the included studies was severity of depression (6/12). The Patient Health Questionnaire (PHQ-9) was the most used outcome measure in the included studies (4/12). The follow-up periods were 2 weeks (6/12), 4 weeks (6/12), 6 weeks (1/12), and 12 weeks (1/12). Characteristics of the comparators and measured outcomes in each included study are presented in Multimedia Appendix 6.

### Risk of Bias in the Included Studies

**Figure 2:**
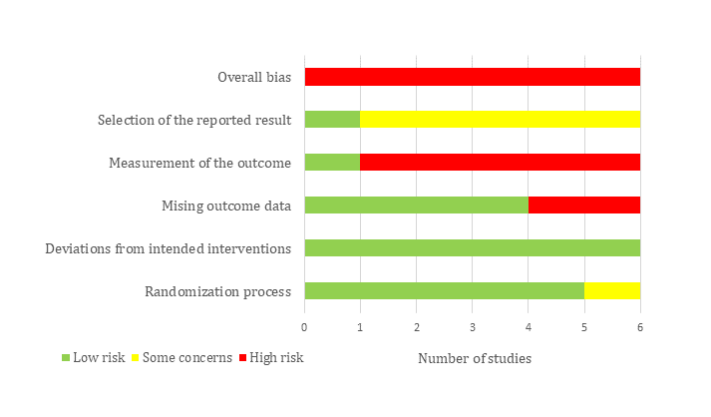
Risk of bias graph for randomized controlled trials, showing the review authors’ judgments about each risk of bias item.

Most of the RCTs (5/6) used an appropriate random allocation sequence, concealed that allocation sequence, and had comparable groups. These studies were rated as having a low risk of bias in the randomization process (Figure 2). Although participants, carers, and people delivering the interventions were aware of the assigned intervention during the trial in most studies (this may be normal due to the nature of the intervention), there were no deviations from the intended intervention because of the experimental context in all studies. Given the lack of deviation and using an appropriate analysis to estimate the effect of assignment to the intervention, a risk of bias due to deviations from the intended interventions was considered low for all studies (Figure 2). The domain of missing outcome data was judged as having a low risk of bias in 4 studies while it was rated as having a high risk of bias in the remaining 2 studies due to a high attrition rate, lack of analysis methods used to correct for bias, and presence of differences between intervention groups in the proportions of missing outcome data.

Although the methods of measuring the outcomes were appropriate and they were comparable between intervention groups (in terms of tools, thresholds, and timing), the risk of bias in the measurement of the outcome was high in 5 studies (Figure 2). This is attributed to the fact that assessors of the outcome were aware of the intervention received by study participants and this knowledge could affect the outcome assessment in those 5 studies. Five studies were judged to raise some concerns in the selection of the reported result (Figure 2). This judgment was due to a discrepancy between studies and their protocols in planned outcome measurements and analyses, unavailability of their protocols, or insufficient details in their protocols regarding outcome measurements and analyses. The overall risk of bias was rated as high for all studies because 5 studies were assessed as high risk in at least one domain, while the remaining study had some concerns in two domains. Multimedia Appendix 7 shows the reviewers’ judgments about each “risk of bias” domain for each included RCT.

**Figure 3:**
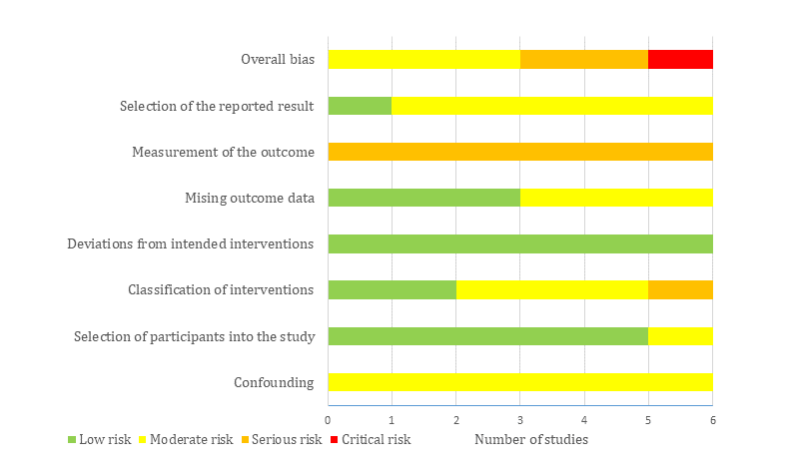
Risk of bias graph for quasiexperiements, showing the review authors’ judgments about each risk of bias item.

There was moderate risk of bias due to confounding in all quasiexperimental studies (Figure 3). This judgment was based on a potential for confounding of the effect of intervention in all studies, and it was not clear whether authors in all studies used an appropriate analysis method to control for all confounding domains. The selection of participants was not based on participant characteristics observed after the start of the intervention in 5 studies, and the start of follow-up and start of intervention coincided for most participants in all studies. Accordingly, the “risk of bias due to selection of participant” domain was judged as low in the 5 studies (Figure 3). Although all studies clearly defined the intervention groups at the start of the intervention, it was not clear whether classification of intervention status could be affected by knowledge of the outcome in 3 studies. Therefore, the risk of bias in the classification of the interventions was rated as high in those 3 studies. Further, the risk of bias in this domain was judged as serious in one study, as the classification of the intervention status could be affected by knowledge of the outcome in that study.

Given that there were no deviations from the intended intervention beyond what would be expected in usual practice in all studies, the risk of bias from the deviations from the intended interventions was considered low in all studies (Figure 3). The risk of bias due to missing outcome data was judged as low in 3 studies while it was rated as moderate in the remaining 3 studies due to availability of less than 95% of the participants’ data. The risk of bias in the measurement of the outcomes was serious in all studies (Figure 3); assessors of the outcome were aware of the intervention received by study participants, and this could affect the assessment of outcomes. In 5 studies, there was moderate risk of bias in the selection of the reported results (Figure 3); this is because there were insufficient details about the analyses used in the study. While the overall risk of bias was rated as critical in 1 study, it was judged as moderate and serious in 3 and 2 studies, respectively. Multimedia Appendix 8 shows the reviewers’ judgments about each “risk of bias” domain for each included quasiexperiment.

### Results of Studies

#### Depression

Half of the included studies (6/12) examined the effect of using chatbots on the severity of depression [27-32]. Of these 6 studies, 4 studies were RCTs [27-30], and the remaining 2 studies were pretest-posttest quasiexperiments [31,32]. Four studies were conducted in the United States [28-30,32], and each of the 2 remaining studies was conducted in multiple countries [27,31]. The severity of depression was measured using PHQ-9 [28,29,31,32], Beck Depression Inventory II [27], and Hospital Anxiety and Depression Scale [30].

**Figure 4:**
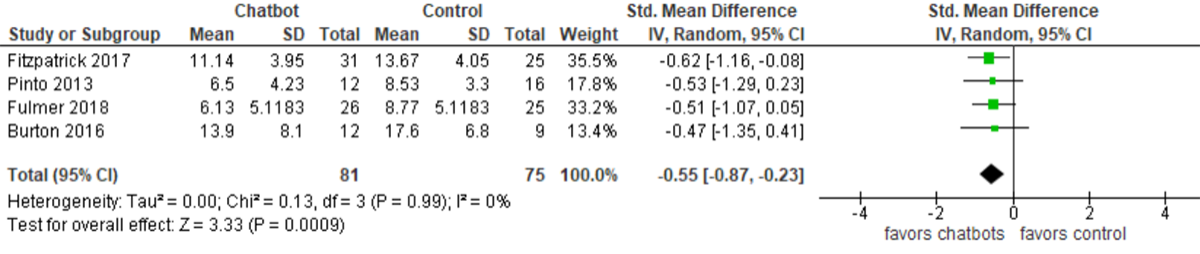
Forest plot of the 4 studies assessing the effect of using chatbots on the severity of depression.

We meta-analyzed the results of only 4 RCTs. However, the results of the 2 quasiexperiments were synthesized narratively because such a study design has a greater risk of bias than RCTs, and some data required for the meta-analysis was missing from 1 of the 2 studies. The meta-analysis showed a statistically significant difference (*P*<.001) favoring chatbots over treatment as usual or information on the severity of depression (SMD –0.55, 95% CI –0.87 to –0.23; Figure 4). However, this difference was not clinically important, as the total effect (–0.55) was within the boundaries of a minimal clinically important difference (–4.7 to 4.7); the boundaries of a minimal clinically important difference for this outcome was calculated as ±0.5 times the median SD of the control arms of the studies at baseline. The heterogeneity of the evidence was not a concern (*P*=.99; I^2^= 0%). The quality of the evidence was low because it was downgraded by 2 levels for a high risk of bias (Multimedia Appendix 9).

Of the 2 quasiexperiments that measured depression, 1 study concluded that the severity of depression decreased significantly postintervention in the high user (*P*<.001) and low user (*P*=.01) groups [31]. Further, the improvement in depression was significantly higher in the high user group than in the low user group (*P*=.03). The second study found a statistically significant decrease in the severity of depression after the intervention (mean 9.78) compared to before the intervention (mean 13.03) [32].

#### Anxiety

Of the 12 studies, 3 studies assessed the influence of using chatbots on the severity of anxiety [28,29,32]. All studies were conducted in the United States. The severity of anxiety was measured using the Generalized Anxiety Disorder scale [28,29] and Overall Anxiety Severity and Impairment Scale [32]. While 2 studies were RCTs [28,29], the third study was a pretest-posttest quasiexperiment [32]. In contrast to the 2 RCTs, the quasiexperiment was a one-arm trial [32]. For this reason, only the findings of the 2 RCTs were meta-analyzed.

**Figure 5:**
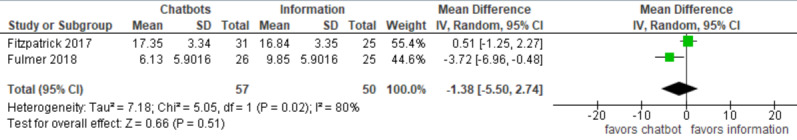
Forest plot of the 2 studies assessing the effect of using chatbots on the severity of anxiety.

As shown in Figure 5, no statistically significant difference (*P*=.55) in the severity of anxiety was found between those allocated to receive the chatbot intervention compared to those receiving information only (MD –1.38, 95% CI –5.5 to 2.74). There was substantial heterogeneity (*P*=.02; I^2^=80%). The quality of the evidence was very low because it was downgraded by 3 levels due to a high risk of bias and heterogeneity (Multimedia Appendix 9). The third study concluded that there was a statistically significant decrease in anxiety level among participants after using chatbots (mean 10.45 versus 7.89) [32].

#### Positive and Negative Affect

The effect of using chatbots on positive and negative affect, which is an indicator of depression and anxiety, was examined in 2 studies [28,29]. Both studies were RCTs conducted in the United States [28,29]. The outcome in the 3 studies was measured using the Positive and Negative Affect Schedule. Meta-analysis could not be executed as only 1 study reported enough data for the analysis [28].

The first study found no statistically significant difference between chatbot use and information on positive affect (*P*=.71) and negative affect (*P*=.91) [28]. In contrast, Fulmer et al [29] found a statistically significant difference favoring chatbot use over information on positive and negative affect at the 2-week follow-up (*P*=.03).

#### Subjective Psychological Wellbeing

The effect of using chatbots on subjective psychological wellbeing was examined by 3 studies [33-35]. Those studies were conducted in Sweden, Turkey, and Japan, respectively [33-35]. Of the 3 studies, 2 studies were quasiexperiments [34,35], and the remaining study was an RCT [33]. The Flourishing Scale was used to measure subjective psychological wellbeing in 2 studies [33,34], whereas the WHO-5 Well-Being Index was used by the third study [35]. Given that the high risk associated with quasiexperiments and availability of only one RCT, the results of the 3 studies were synthesized narratively.

In the first study [33], the intention-to-treat analysis showed that subjective psychological wellbeing was not statistically different (*P*=.97) after treatment between the chatbot (mean 45.14) and waiting list (mean 45.07) groups. Further, when analyzing data from only the participants who adhered to the intervention, there was no statistically significant difference (*P*=.72) between the chatbot and waiting list groups on subjective psychological wellbeing after treatment (mean 45.07 versus 45.85) [33]. The second study demonstrated a slight improvement in subjective psychological wellbeing after using chatbots, but this improvement was not statistically significant (*P*=.06) [34]. Similarly, the third study found no statistically significant difference (*P*=.32) between the chatbot and control groups on subjective psychological wellbeing after treatment [35].

#### Psychological Distress

The influence of using chatbots on psychological distress was examined by 2 studies, conducted in Japan and Australia [35,36]. Distress was measured using the Kessler Psychological Distress Scale. While 1 study was a one-group quasiexperiment [36], the other study was a two-group quasiexperiment [35]. Therefore, a narrative approach was used to analyze their results.

According to Suganuma et al [35], there was a statistically significant difference (*P*=.005) favoring chatbot use (mean 21.65) over no intervention (mean 23.97) on distress levels after treatment. Further, there was a statistically significant improvement in distress level among the chatbot group after treatment (mean 21.65) compared with before treatment (mean 23.58). Likewise, the other study found a statistically significant decrease (*P*<.001) in distress from a pre-intervention score of 33.27 to a post-intervention score of 28.90 [36].

#### Stress

Stress was an outcome in 2 studies [33,37]. The first was an RCT conducted in Sweden [33], and the second was a quasiexperimental study conducted in China [37]. The Perceived Stress Scale was utilized to measure stress in both studies. A meta-analysis was not carried out for this outcome as 1 study [37] did not report data required for the analysis.

Ly and colleagues [33] found a statistically significant difference favoring chatbots over the waiting list on stress when they analyzed data from all participants (*P*=.03) and from those who only adhered to the intervention (*P*=.01). Huang et al [37] concluded that stress status improved over time when using a chatbot.

#### Acrophobia

The effect of using chatbots on acrophobia (ie, fear of height) was examined by 1 RCT conducted in the United Kingdom [38]. The outcome was measured using two tools: Heights Interpretation Questionnaire and Acrophobia Questionnaire. Compared with participants who received usual care, the chatbot significantly decreased the severity of acrophobia as measured by both tools at 2-week and 4-week follow-ups (*P*<.001) [38].

#### Safety

Safety of chatbots was assessed in 2 RCTs [30,38]. While 1 study was conducted in the United States [30], the other study was conducted in the United Kingdom [38]. The former study concluded that the chatbot was safe because users did not report any harm, distress, adverse events, or worsening of depressive symptoms resulting from using the chatbot during the study [30]. Similarly, Freeman et al [38] concluded that the chatbot was safe because no serious adverse events (eg, suicide attempts, death, serious violent incidents) or discomfort caused by the chatbot were reported.

## Discussion

### Principal Findings

This study systematically reviewed the evidence regarding the effectiveness and safety of using chatbots to improve mental health. We identified 12 studies examining the effect of using chatbots on 8 outcomes. For the first outcome (depression), low-quality evidence from 4 RCTs showed a statistically significant difference favoring chatbots over treatment as usual or information on the severity of depression, but this difference was not clinically important. Two quasiexperiments concluded that the level of depression decreased after using chatbots. As evidence from the 2 studies was synthesized narratively, we could not identify whether this decrease in depression was clinically important. Findings in the 2 studies may be affected by serious bias in the measurement of outcomes. Given that no reviews assessed the effectiveness of chatbots in mental health, the results were compared with other reviews regarding similar interventions (ie, internet-based psychotherapeutic interventions). The overall effect on depression in this review (–0.55) was comparable to other reviews. Specifically, while the overall effect of internet-based and computerized psychological interventions of depression without therapist support was 0.25 (95% CI 0.14-0.35) in a meta-analysis conducted by Andersson and Cuijpers [39], another meta-analysis showed that the total effect of internet-based psychotherapeutic interventions of depression was 0.32 [40].

With regards to anxiety, very low-quality evidence from 2 RCTs showed no statistically significant difference between chatbots and information on the severity of anxiety. In contrast, one quasiexperiment concluded that anxiety levels considerably decreased after using chatbots. These contradictory findings may be attributed to 2 reasons. First, pretest-posttest quasiexperiments are not as reliable as RCTs for finding the effect of an intervention due to low internal validity resulting from selection bias [35,41]. Second, in contrast to the 2 RCTs, the chatbot in the quasiexperiment [32] contained a virtual representation (ie, embodiment), which enables chatbots to communicate with users verbally and nonverbally (through body movements and facial expressions). It is purported that embodiment makes conversations with chatbots more empathetic and facilitates effective rapport with users [19,42,43]. Results of the meta-analyses in this review and another review related to smartphone mental health interventions were contradictory. A meta-analysis of 9 RCTs showed a considerable reduction in the anxiety level after using smartphone mental health interventions compared to no intervention (SMD 0.325, 95% CI 0.17-0.48) [44]. These conflicting results may result from either differences in interventions (chatbots versus different mobile interventions) in both reviews or the number of meta-analyzed studies (2 versus 9).

Findings regarding the effect of chatbots on positive and negative affect were conflicting. While one study concluded that chatbots improved the positive and negative affect at the 2-week follow-up [29], another study did not find any significant influence of chatbots at the 2-week follow-up [28]. Although the 2 studies were very homogenous in terms of study design, sample characteristics, comparator characteristics, and outcome measures, they were different in the type of chatbots and data analysis, and these differences may have led to contradictory findings. Specifically, the chatbot in the first study [29] was more advanced than the one in the second study [28]; it depended on artificial intelligence and machine learning to generate responses to users, and this makes it more humanlike and lets users feel more socially connected [5]. With regards to the second difference, while the first study assessed the effect of the chatbot on positive and negative affect together [29], the other study examined the effect of the chatbot on positive affect and negative affect separately [28].

A narrative synthesis of 3 studies showed no statistically significant difference between chatbots and control group on subjective psychological wellbeing. The justification for the nonsignificant difference is the use of a nonclinical sample in the 3 studies. In other words, as participants already had good psychological wellbeing, the effect of using chatbots may be less likely to be significant.

According to the 2 studies synthesized in a narrative approach, chatbots significantly decreased the levels of distress. Both studies had a high risk of bias; therefore, this finding should be interpreted with caution. Studies in a similar context reported findings comparable to our findings. To be more precise, an RCT concluded that online chat counselling significantly improved psychological distress over time [45].

In this review, chatbots significantly decreased stress levels over time. Unfortunately, we cannot draw a definitive conclusion regarding the effect of chatbots due to the high risk of bias in the evidence.

Chatbots were effective in decreasing the severity of acrophobia according to one RCT. The effect size of chatbots on acrophobia in this RCT [38] was substantially higher than the total effect size of therapist-assisted exposure treatment on phobias reported by a meta-analysis (2.0 versus 1.1) [46]. This indicates that chatbots may be equivalent to, if not better, exposure treatment delivered by a therapist in treating phobias.

Of the 2 RCTs measuring the safety of chatbots, both concluded that chatbots are safe for use in mental health, as no adverse events or harm were reported when chatbots were used to treat users with depression and acrophobia. However, this evidence is not sufficient to conclude that chatbots are safe, given the high risk of bias in the 2 studies.

### Strengths and Limitations

#### Strengths

This study is the first review of the literature that assessed the effectiveness and safety of chatbots in mental health. The findings are of importance for users, providers, policymakers, and researchers. This review was developed, executed, and reported according to the Preferred Reporting Items for Systematic Reviews and Meta-Analyses statement [20]. Accordingly, this enabled us to produce a high-quality review.

In this review, the most popular databases in health and information technology were used to run the most sensitive search possible. The review minimized the risk of publication bias as much as possible through searching Google Scholar and conducting backward and forward reference list checking to identify grey literature. The search was not restricted to a certain type of chatbots, comparators, outcomes, year of publication, nor country of publication, and this makes the review more comprehensive.

To reduce selection bias, two reviewers independently selected studies, extracted data, and assessed the risk of bias in the included studies and quality of the evidence. Agreement between reviewers was very good, except for the assessment of the risk of bias (which was good). When possible, findings of the included studies were meta-analyzed; thereby, we were able to increase the power of the studies and improve the estimates of the likely size of effect of chatbots on a variety of mental health outcomes.

#### Limitations

The intervention of interest in this review was restricted to chatbots that work within standalone software or via a web browser (but not robotics, serious games, SMS, nor telephones). Further, we excluded studies that contained chatbots controlled by human operators. Accordingly, this review cannot comment on the effectiveness of chatbots that involve human-generated content or those that use alternative modes of delivery. It was necessary to apply those restrictions because these features are not part of ordinary chatbots. For this reason, 3 previous reviews about chatbots applied these restrictions [5,13,19].

Owing to practical constraints, the search was restricted to English studies. Therefore, it is likely that we missed some non-English studies. The overall risk of bias was high in most of the included studies. The quality of evidence in the meta-analyses ranged from very low to low. Accordingly, the high risk of bias and low quality of evidence may reduce the validity of the findings and their generalizability.

Ideally, the difference between pre-intervention and post-intervention data for each group should be used in a meta-analysis [47]. However, we used only post-intervention data in each group for the meta-analysis because studies did not report enough data (eg, change in SD or SE of the mean between the pre-intervention and post-intervention for each group). In this review, it was possible to meta-analyze pre-intervention and post-intervention data from one-group trials (ie, did not include comparison groups). However, such analysis was not carried out in this review as such trials are very vulnerable to several threats of internal validity, such as maturation threat, instrumentation threat, regression threat, and history threat [41,48].

### Practical and Research Implications

#### Practical Implications

Although this review found that chatbots may improve depression, distress, stress, and acrophobia, definitive conclusions regarding those results could not be drawn due to the high risk of bias in the included studies, low quality of evidence, lack of studies assessing each outcome, small sample size in the included studies, and contradictions in results of some included studies. For this reason, results should be viewed with caution by users, health care providers, caregivers, policymakers, and chatbot developers.

Given the weak and conflicting evidence found in this review, users should not use chatbots as a replacement for mental health professionals. Instead, health professionals should consider offering chatbots as an adjunct to already available interventions to encourage individuals to seek medical advice where appropriate and as a signpost to available support and treatment.

Most chatbots in this review were implemented in developed counties. People in developing countries may be more in need of chatbots than those in developed countries given that developing countries have a greater shortage of mental health professionals than developed countries (0.1 per 1,000,000 people vs 9 per 100,000 people) [7,8]. System developers should consider implementing more chatbots in developing countries.

Two-thirds of the chatbots in this review used predefined rules and decision trees to generate their responses, while the remaining chatbots used artificial intelligence. In contrast to rule-based chatbots, artificial intelligence chatbots can generate responses to complicated queries and enable users to control the conversation [13]. Artificial intelligence chatbots can exhibit more empathetic behaviors and humanlike filler language than rule-based chatbots [19]. This may make artificial intelligence chatbots more effective in building rapport with users, thereby improving their mental health [42]. It could be argued that artificial intelligence chatbots are more prone to errors than rule-based chatbots, but these errors can be minimized and diminished by extensive training and greater use [49]. Accordingly, we recommend developers concentrate efforts around artificial intelligence chatbots to improve the effectiveness.

#### Research Implications

This review showed that there is a lack of evidence assessing the effectiveness and safety of chatbots. Accordingly, we encourage researchers to conduct more studies in this area. Further, they should undertake more studies in developing countries and recruit large, clinical samples given the lack of such evidence, as found in the current review.

The overall risk of bias was high in most included studies mainly due to issues in the measurement of the outcomes, selection of the reported result, and confounding. Future studies should follow recommended guidelines or tools (eg, RoB 2 and ROBINS-I) when conducting and reporting their studies in order to avoid such biases.

Due to poor reporting practices, we were unable to include many studies in the meta-analysis. As well as encouraging more high-level studies (ie, RCTs), there is a need for authors to be more consistent in their reporting of trial outcomes. For example, in our review, many studies failed to report basic descriptive statistics such as mean, SD, and sample size. Ensuring studies adhere to accepted guidelines for reporting RCTs (eg, CONSORT-EHEALTH [50]) would be of considerable benefit to the field.

In the current review, the comparators in all two-group trials were either no intervention or education. For those outcomes that hold promise (eg, depression, distress, and acrophobia), we encourage researchers to compare chatbots with other active interventions such as asynchronous electronic interventions or other types of chatbots (eg, rule-based chatbots versus artificial intelligence chatbots or embodied chatbots versus non-embodied chatbots).

According to a scoping review conducted by Abd-alrazaq et al [13], chatbots are used for many mental disorders, such as autism, post-traumatic stress disorder, substance use disorders, schizophrenia, and dementia. The current review did not find any study assessing the effectiveness or safety of chatbots used for these disorders. This highlights a pressing need to examine the effectiveness and safety of chatbots targeting patients with autism, post-traumatic stress disorder, substance use disorders, schizophrenia, and dementia.

As this review focused on the effectiveness and safety of chatbots, we excluded many studies that assessed the usability and acceptance of chatbots in mental health. Given that usability and acceptance of technology are considered important factors for their successful implementation, the evidence regarding those outcomes should be summarized through systematic reviews.

The current review identified heterogeneity in the tools used to measure the same outcomes and in the research design. For instance, severity of depression was measured using PHQ-9, Beck Depression Inventory II, or Hospital Anxiety and Depression Scale. Further, while some studies assessed outcomes before and after interventions, other studies examined them only after interventions. The field would benefit from future studies using a common set of outcome measures to ease comparison and interpretation of results between studies. Only one study assessed the long-term effectiveness and safety of chatbots, where participants were followed for 12 weeks. The effectiveness and safety outcomes of chatbots may be different when considering long-term, relative to short-term, findings; it is essential to assess long-term outcomes.

### Conclusion

Although the included studies showed that chatbots may be safe and improve depression, distress, stress, and acrophobia, definitive conclusions regarding the effectiveness and safety of chatbots could not be drawn in this review for several reasons. First, the statistically significant difference between chatbots and other interventions on the severity of depression was not clinically important. Second, the risk of bias was high in most included studies, and the quality of the meta-analyzed evidence ranged from very low to low. Third, the evidence for each outcome came from only a few studies that also had small sample sizes. Fourth, studies showed conflicting results for some outcomes (ie, anxiety and positive and negative affect). Researchers should avoid shortcomings in the study designs reported in this review. Health care providers should consider offering chatbots as an adjunct to already available interventions.

## Appendix

Multimedia Appendix 1 PRISMA checklist.

Multimedia Appendix 2 Search strings utilized for searching each bibliographic database.

Multimedia Appendix 3 Data extraction form.

Multimedia Appendix 4 Characteristics of each included study.

Multimedia Appendix 5 Characteristics of the intervention in each study.

Multimedia Appendix 6 Characteristics of comparators and measured outcomes in each included study.

Multimedia Appendix 7 Reviewers’ judgements about each “risk of bias” domain for each included RCT.

Multimedia Appendix 8 Reviewers’ judgements about each “risk of bias” domain for each included quasi-experiment.

Multimedia Appendix 9 GRADE evidence profile.

## Abbreviations

AQ: Acrophobia Questionnaire.
BDI-2: Beck Depression Inventory II.
FS: Flourishing Scale.
GAD-7: Generalized Anxiety Disorder scale.
HAD-S: Hospital Anxiety and Depression Scale.
HIQ: Heights Interpretation Questionnaire.
K10: Kessler Psychological Distress Scale.
MD: mean difference.
MCID: Minimal clinically important difference
OASIS: Overall Anxiety Severity and Impairment Scale.
PANAS: Positive and Negative Affect Schedule.
PHQ-9: Patient Health Questionnaire.
PSS-10: Perceived Stress Scale.
RCT: randomized controlled trial.
RoB 2: Risk-of-Bias 2.
ROBINS-I: Risk Of Bias In Non-randomized Studies – of Interventions.
SMD: standardized mean difference.
WHO-5-J: World Health Organization-5 Well-Being Index.
