# Supplementary material for: Effectiveness and Safety of Using Chatbots to Improve Mental Health: Systematic Review and Meta-Analysis
Source: J Med Internet Res. 2020 Jul 13;22(7):e16021. doi: 10.2196/16021 (PMC7385637; doi:10.2196/16021)
Supplement: Multimedia Appendix 3 [file jmir_v22i7e16021_app3.docx]

| **Concept** | **Definition** |
| --- | --- |
| **Study Characteristics** |  |
| Author | The first author of the study. |
| Year of Publication | The year in which the study was published. |
| Country of publication | The country where the study was published. |
| Type of publication | The medium in which the study was published (e.g. conference proceedings, journal, thesis). |
| Study design | The research method that the study used to collect the data (e.g. RCTs and quasi-experiments) |
| Study aim | What the study aimed to find out. |
| **Population characteristics** |  |
| Number of participants | Number of people who participated in the study. |
| Mean age | The average age of participants. |
| Gender (male) | Percentage of males in the sample. |
| Health condition | Health status of participants (e.g. clinical or non-clinical sample). |
| Recruitment Setting | Place where participants were recruited (i.e. clinical, educational, community settings). |
| **Intervention characteristics** |  |
| Chatbot name | Name of the chatbot. |
| Chatbot aim | What it is that the chatbot aims to achieve (therapy, education, counselling, self-management, screening, diagnosing) |
| Platform | The platform in which the chatbot was implemented (i.e. stand-alone software, web-based, or multimodal platform). |
| Response generation | The method of processing inputs and generating responses:   1. Rule-based: chatbots that answer questions based on some predefined rules on which it is trained on. 2. Artificial intelligence-based: chatbots that use some machine learning and natural language processing to understand the context and intent of a question and to respond to it. |
| Dialogue initiative | Who leads the conversation: user, chatbot, both? |
| Input modality | How the user interacts with the chatbot: spoken language (via microphones), written language (via keyboards and mouse), visual language (facial expression and body language via camera or Kinect). |
| Output modality | How the chatbot interacts with the user: spoken language (via speakers), written language (via text on the screen), visual language (facial expression and body language via embodiment). |
| Embodiment | Whether there is a virtual representation (e.g. avatar or virtual human) shown on the screen |
| Targeted disorder | The disorder that the chatbot was designed for. |
| **Comparator** **Characteristics** |  |
| Comparator | What is the comparator (e.g. usual care, waiting list, not intervention, giving information)? |
| **Outcome characteristics** |  |
| Measured outcome | What is the outcome that the study measured?   1. Effectiveness: Severity or frequency of any mental disorder, and psychological well-being. 2. Safety: Adverse events, admissions to psychiatric settings, deaths |
| Outcome measure | What is the tool used for measuring the outcome? |
| Follow-up period | When was the outcome measured? |
| **Findings** |  |
| Results of the study | Any reported statistics related to the outcome of interest, e.g. numbers, means, mean differences, standard deviations, confidence intervals, and *P*-values. |
