# Supplementary material for: Effectiveness and Safety of Using Chatbots to Improve Mental Health: Systematic Review and Meta-Analysis
Source: J Med Internet Res. 2020 Jul 13;22(7):e16021. doi: 10.2196/16021 (PMC7385637; doi:10.2196/16021)
Supplement: Multimedia Appendix 4 [file jmir_v22i7e16021_app4.docx]

| Author ^ID^ | Country | Publication type | Study design | Sample size | Mean age | Sex (male) | Sample type | Recruitment Setting |
| --- | --- | --- | --- | --- | --- | --- | --- | --- |
| Burton (2016)^1^ | Romania, Spain, Scotland | Journal | RCT | 27 | 39 | 33% | Clinical | Clinical |
| Fitzpatrick (2017)^2^ | USA | Journal | RCT | 70 | 22.2 | 33% | Clinical | Clinical |
| Fulmer (2018)^3^ | USA | Journal | RCT | 75 | 22.9 | 30% | Non-clinical | Educational |
| Pinto (2015)^4^ | USA | Journal | RCT | 28 | 22 | 33% | Clinical | Community |
| Inkster (2018)^5^ | Global population | Journal | Quasi-experiment | 129 | - | - | Non-clinical | Community |
| Schroeder (2018)^6^ | USA | Conference proc. | Quasi-experiment | 73 | 37.3 | 9.5% | Clinical | Clinical |
| Ly (2017)^7^ | Sweden | Journal | RCT | 28 | 26.2 | 46.4% | Non-clinical | Educational & Community |
| Demirci (2018)^8^ | Turkey | Dissertation | Quasi-experiment | 16 | 28 | 56% | Non-clinical | Educational |
| Suganuma (2018)^9^ | Japan | Journal | Quasi-experiment | 454 | 38.1 | 30% | Non-clinical | Community |
| Luerssen (2018)^10^ | Australia | Conference proc. | Quasi-experiment | 163 | 32 | - | Clinical | Community |
| Huang (2015)^11^ | China | Conference proc. | Quasi-experiment | 10 | - | - | Non-clinical | Educational |
| Freeman (2018)^12^ | UK | Journal | RCT | 100 | 45 | 48% | Clinical | Community |

**Included studies**

1. Burton C, Tatar AS, McKinstry B, Matheson C, Matu S, Moldovan R, et al. Pilot randomised controlled trial of Help4Mood, an embodied virtual agent-based system to support treatment of depression. Journal of Telemedicine and Telecare. 2016 Sep;22(6):348-55. PMID: 2016-40249-004.

2. Fitzpatrick KK, Darcy A, Vierhile M. Delivering Cognitive Behavior Therapy to Young Adults With Symptoms of Depression and Anxiety Using a Fully Automated Conversational Agent (Woebot): A Randomized Controlled Trial. JMIR Ment Health. 2017 Jun 6;4(2):e19. PMID: 28588005. doi: 10.2196/mental.7785.

3. Fulmer R, Joerin A, Gentile B, Lakerink L, Rauws M. Using Psychological Artificial Intelligence (Tess) to Relieve Symptoms of Depression and Anxiety: Randomized Controlled Trial. JMIR Ment Health. 2018 Dec 13;5(4):e64. PMID: 30545815. doi: 10.2196/mental.9782.

4. Pinto MD, Greenblatt AM, Hickman RL, Rice HM, Thomas TL, Clochesy JM. Assessing the critical parameters of eSMART-MH: A promising avatar-based digital therapeutic intervention to reduce depressive symptoms. Perspectives in Psychiatric Care. 2015 Jul;52(3):157-68. PMID: 2016-32710-003.

5. Inkster B, Sarda S, Subramanian V. An Empathy-Driven, Conversational Artificial Intelligence Agent (Wysa) for Digital Mental Well-Being: Real-World Data Evaluation Mixed-Methods Study. JMIR Mhealth Uhealth. 2018 Nov 23;6(11):e12106. PMID: 30470676. doi: 10.2196/12106.

6. Schroeder J, Wilkes C, Rowan K, Toledo A, Paradiso A, Czerwinski M, et al. Pocket Skills: A Conversational Mobile Web App To Support Dialectical Behavioral Therapy. Proceedings of the 2018 CHI Conference on Human Factors in Computing Systems; Montreal QC, Canada: ACM; 2018.

7. Ly KH, Ly AM, Andersson G. A fully automated conversational agent for promoting mental well-being: A pilot RCT using mixed methods. Internet Interventions. 2017;10:39-46. doi: <http://0-dx.doi.org.wam.leeds.ac.uk/10.1016/j.invent.2017.10.002>.

8. Demirci HM. User experience over time with conversational agents case study of woebot on supporting subjective well-being: Middle East Technical University; 2018.

9. Suganuma S, Sakamoto D, Shimoyama H. An Embodied Conversational Agent for Unguided Internet-Based Cognitive Behavior Therapy in Preventative Mental Health: Feasibility and Acceptability Pilot Trial. JMIR Ment Health. 2018 Jul 31;5(3):e10454. PMID: 30064969. doi: 10.2196/10454.

10. Luerssen MH, Hawke T. Virtual Agents as a Service: Applications in Healthcare. Proceedings of the 18th International Conference on Intelligent Virtual Agents; Sydney, NSW, Australia: ACM; 2018.

11. Huang J, Li Q, Xue Y, Cheng T, Xu S, Jia J, et al., editors. Teenchat: a chatterbot system for sensing and releasing adolescents’ stress. International Conference on Health Information Science; 2015: Springer.

12. Freeman D, Haselton P, Freeman J, Spanlang B, Kishore S, Albery E, et al. Automated psychological therapy using immersive virtual reality for treatment of fear of heights: A single-blind, parallel-group, randomised controlled trial. The Lancet Psychiatry. 2018 Aug;5(8):625-32. PMID: 2018-38160-017.
