# Supplementary material for: Effectiveness and Safety of Using Chatbots to Improve Mental Health: Systematic Review and Meta-Analysis
Source: J Med Internet Res. 2020 Jul 13;22(7):e16021. doi: 10.2196/16021 (PMC7385637; doi:10.2196/16021)
Supplement: Multimedia Appendix 7 [file jmir_v22i7e16021_app7.docx]

**Included studies**

1. Burton C, Tatar AS, McKinstry B, Matheson C, Matu S, Moldovan R, et al. Pilot randomised controlled trial of Help4Mood, an embodied virtual agent-based system to support treatment of depression. Journal of Telemedicine and Telecare. 2016 Sep;22(6):348-55. PMID: 2016-40249-004.

2. Fitzpatrick KK, Darcy A, Vierhile M. Delivering Cognitive Behavior Therapy to Young Adults With Symptoms of Depression and Anxiety Using a Fully Automated Conversational Agent (Woebot): A Randomized Controlled Trial. JMIR Ment Health. 2017 Jun 6;4(2):e19. PMID: 28588005. doi: 10.2196/mental.7785.

3. Fulmer R, Joerin A, Gentile B, Lakerink L, Rauws M. Using Psychological Artificial Intelligence (Tess) to Relieve Symptoms of Depression and Anxiety: Randomized Controlled Trial. JMIR Ment Health. 2018 Dec 13;5(4):e64. PMID: 30545815. doi: 10.2196/mental.9782.

4. Pinto MD, Greenblatt AM, Hickman RL, Rice HM, Thomas TL, Clochesy JM. Assessing the critical parameters of eSMART-MH: A promising avatar-based digital therapeutic intervention to reduce depressive symptoms. Perspectives in Psychiatric Care. 2015 Jul;52(3):157-68. PMID: 2016-32710-003.

7. Ly KH, Ly AM, Andersson G. A fully automated conversational agent for promoting mental well-being: A pilot RCT using mixed methods. Internet Interventions. 2017;10:39-46. doi: <http://0-dx.doi.org.wam.leeds.ac.uk/10.1016/j.invent.2017.10.002>.

12. Freeman D, Haselton P, Freeman J, Spanlang B, Kishore S, Albery E, et al. Automated psychological therapy using immersive virtual reality for treatment of fear of heights: A single-blind, parallel-group, randomised controlled trial. The Lancet Psychiatry. 2018 Aug;5(8):625-32. PMID: 2018-38160-017.
