# Supplementary material for: Effectiveness and Safety of Using Chatbots to Improve Mental Health: Systematic Review and Meta-Analysis
Source: J Med Internet Res. 2020 Jul 13;22(7):e16021. doi: 10.2196/16021 (PMC7385637; doi:10.2196/16021)
Supplement: Multimedia Appendix 8 [file jmir_v22i7e16021_app8.docx]

| ID | Confounding | Selection of participants into the study | Classification of interventions | Deviations from intended interventions | Missing outcome data | Measurement of the outcomes | Selection of the reported result | Overall bias |  |  |  |
| --- | --- | --- | --- | --- | --- | --- | --- | --- | --- | --- | --- |
| 5 |  |  |  |  |  |  |  |  |  | **Key** | |
| 6 |  |  |  |  |  |  |  |  |  |  | Low risk |
| 8 |  |  |  |  |  |  |  |  |  |  | Moderate risk |
| 9 |  |  |  |  |  |  |  |  |  |  | Serious risk |
| 10 |  |  |  |  |  |  |  |  |  |  | Critical risk |
| 11 |  |  |  |  |  |  |  |  |  |  |  |

**Included studies**

5. Inkster B, Sarda S, Subramanian V. An Empathy-Driven, Conversational Artificial Intelligence Agent (Wysa) for Digital Mental Well-Being: Real-World Data Evaluation Mixed-Methods Study. JMIR Mhealth Uhealth. 2018 Nov 23;6(11):e12106. PMID: 30470676. doi: 10.2196/12106.

6. Schroeder J, Wilkes C, Rowan K, Toledo A, Paradiso A, Czerwinski M, et al. Pocket Skills: A Conversational Mobile Web App To Support Dialectical Behavioral Therapy. Proceedings of the 2018 CHI Conference on Human Factors in Computing Systems; Montreal QC, Canada: ACM; 2018.

8. Demirci HM. User experience over time with conversational agents case study of woebot on supporting subjective well-being: Middle East Technical University; 2018.

9. Suganuma S, Sakamoto D, Shimoyama H. An Embodied Conversational Agent for Unguided Internet-Based Cognitive Behavior Therapy in Preventative Mental Health: Feasibility and Acceptability Pilot Trial. JMIR Ment Health. 2018 Jul 31;5(3):e10454. PMID: 30064969. doi: 10.2196/10454.

10. Luerssen MH, Hawke T. Virtual Agents as a Service: Applications in Healthcare. Proceedings of the 18th International Conference on Intelligent Virtual Agents; Sydney, NSW, Australia: ACM; 2018.

11. Huang J, Li Q, Xue Y, Cheng T, Xu S, Jia J, et al., editors. Teenchat: a chatterbot system for sensing and releasing adolescents’ stress. International Conference on Health Information Science; 2015: Springer.
