# Supplementary material for: Effectiveness and Safety of Using Chatbots to Improve Mental Health: Systematic Review and Meta-Analysis
Source: J Med Internet Res. 2020 Jul 13;22(7):e16021. doi: 10.2196/16021 (PMC7385637; doi:10.2196/16021)
Supplement: Multimedia Appendix 9 [file jmir_v22i7e16021_app9.docx]

| **GRADE Profile for comparison of chatbots to usual treatment or information** | | | | | | | | | | | |
| --- | --- | --- | --- | --- | --- | --- | --- | --- | --- | --- | --- |
| **Certainty assessment** | | | | | | | **Summary of findings** | | | | |
| **No of participants (studies) Follow-up** | **Risk of bias** | **Inconsistency** | **Indirectness** | **Imprecision** | **Publication bias** | **Overall certainty of evidence** | **Study event rates (%)** | | **Relative effect (95% CI)** | **Anticipated absolute effects** | |
|  |  |  |  |  |  |  | **With Usual care** | **With Chatbots** |  | **Risk with Usual care** | **Risk difference with Chatbots** |
| **Severity of depression (follow up: range 2 weeks to 12 weeks; assessed with: Patient Health Questionnaire (PHQ-9), Beck Depression Inventory II (BDI-2), or Hospital Anxiety and Depression Scale (HAD-S))** | | | | | | | | | | | |
| 156 (4 RCTs) | very serious ^a^ | not serious | not serious | not serious | none | ⨁⨁◯◯ LOW | 75 | 81 | - | - | SMD **0.55 lower** (0.87 lower to 0.23 lower) |
| **Severity of anxiety (follow up: mean 2 weeks; assessed with: Generalized Anxiety Disorder scale (GAD-7); Scale from: 0 to 21)** | | | | | | | | | | | |
| 107 (2 RCTs) | very serious ^b^ | very serious ^c^ | not serious | serious ^d,e^ | none | ⨁◯◯◯ VERY LOW | 50 | 57 | - |  | MD **1.24 lower** (5.33 lower to 2.84 higher) |

**CI:** Confidence interval; **SMD:** Standardized mean difference; **MD:** Mean difference

**Explanations**

a. Evidence downgraded by 2 levels because the overall risk of bias for the 4 studies was rated as high mainly due to issues in measurements of the outcome, selection of the reported results, and missing outcome data.

b. Evidence downgraded by 2 levels because the overall risk of bias for the 2 studies was rated as high mainly due to issues in measurements of the outcome, selection of the reported results, and missing outcome data.

c. Evidence downgraded by 2 as P<0.05 and I square=74%, indicating high heterogeneity.

d. Evidence downgraded by 1 level because 95% CI crosses one MIDs for this outcome.

e. MID for this outcome, calculated as + and - 0.5 times the mean SD of the control arm of each study at baseline, is +/- 4.09.
